# Evaluation of rheological properties of pork myofibrillar protein gel and physicochemical and textural properties of low-fat model sausages containing various levels of chickpea powder dried by different methods

**DOI:** 10.5713/ab.25.0164

**Published:** 2025-08-12

**Authors:** Min Jae Kim, Koo Bok Chin

**Affiliations:** 1 Department of Animal Science, Chonnam National University, Gwangju, Korea

**Keywords:** Chickpea (*Cicer arietinum* L.), Drying Method, Low-fat Sausage, Myofibrillar Protein

## Abstract

**Objective:**

This study evaluated the rheological properties of pork myofibrillar protein (MP) gels, as well as the physicochemical and textural properties of low-fat model sausages (LFMS) formulated with various levels of chickpea powder (CPP) processed via freeze-drying (FCP) or oven-drying (OCP).

**Methods:**

Pork MP gels and LFMS were prepared with varying concentrations (0%–1.5%) of CPP, either as FCP or OCP. Viscosity, cooking yield, gel strength, protein surface hydrophobicity, and sulfhydryl group levels were analyzed, in conjunction with sodium dodecyl sulfate–polyacrylamide gel electrophoresis (SDS-PAGE) and low-vacuum scanning electron microscopy (LV-SEM) investigations, to evaluate the rheological properties and protein structural changes in MP gels after CPP addition. Additionally, cooking loss, expressible moisture, texture profile, SDS-PAGE, and LV-SEM analyses were conducted to assess the physicochemical and textural properties of LFMS containing CPP. The data were analyzed using one-way and two-way ANOVA, followed by Duncan’s multiple range test (p<0.05) to determine significant differences.

**Results:**

Increases in CPP concentration enhanced MP gel viscosity, cooking yield, and gel strength; 1.5% CPP yielded optimal water retention and structural integrity. CPP reduced protein surface hydrophobicity and sulfhydryl content while increasing disulfide bond formation, indicating improved gel network formation. SDS-PAGE confirmed myosin heavy chain reduction and the formation of higher-molecular-weight polymers. In LFMS, CPP reduced cooking loss and expressible moisture; OCP was more effective at higher levels. Texture analysis showed increased hardness and chewiness, whereas cohesiveness and springiness remained unchanged. Microscopy revealed a denser, more uniform structure in sausages containing 1.5% CPP. These changes were correlated with improved water retention and texture.

**Conclusion:**

The findings in this study suggest that CPP, particularly OCP, is a promising functional ingredient for efforts to improve meat product quality by enhancing water retention, gel strength, and texture. CPP also promotes protein polymerization, contributing to a more stable gel network.

## INTRODUCTION

Myofibrillar protein (MP), the primary component of muscle, is responsible for structural stability and functional properties; it primarily consists of structural proteins such as myosin and actin. MP is extracted using salt solutions at concentrations of 0.6 M or higher, a process essential for studying protein solubility and functional characteristics [1]. Myosin, a key component of MP, plays a central role in the gelation process and is closely associated with viscoelastic properties [2]. These viscoelastic properties substantially influence quality indicators such as water-holding capacity (WHC) and texture in meat products [3]. Sun and Holley [4] reported that external protein additives (e.g., egg white, whey protein, soy protein, and sodium caseinate) can enhance MP gel strength, highlighting the potential for additive research to improve meat product quality.

Legumes, such as lentils, chickpeas, and faba beans, are widely cultivated and consumed globally. They are recognized as health foods due to their relatively low cost and high protein content [5,6]. In particular, chickpeas (*Cicer arietinum* L.) contain 23%–24% protein and 36%–41% starch; they constitute an excellent source of fiber, iron, zinc, magnesium, calcium, and vitamins E and B9 [7,8]. Additionally, chickpeas are in high demand as functional ingredients in the food industry due to the functional properties of their components, including protein, starch, polyphenols, and carotenoids [8,9].

Drying, a widely used processing method for fruits and vegetables, is considered an effective technology for extending shelf life by removing moisture and inhibiting microbial growth and water-mediated chemical reactions [10,11]. Oven-drying is a simple and economical method but has disadvantages, including nutrient loss and product quality deterioration. In contrast, freeze-drying, a low-temperature drying method based on ice sublimation, inhibits microbial and enzymatic activity while preserving the original sensory qualities of food. However, it requires high energy consumption [11,12].

Pork sausages are widely consumed due to their excellent nutritional value, convenience, and distinctive flavor [13]. Notably, pork back fat, the primary component of pork sausages, enhances flavor while substantially affecting texture and sensory quality [13]. Due to the increasing prevalences of health concerns and chronic diseases, consumer interest in healthy lifestyles and balanced diets has risen, driving steady demand for nutritious food products [14,15]. In response to this trend, the development of low-fat meat products incorporating non-meat ingredients (e.g., plant-based proteins and carbohydrates) as replacements for animal fat has gained considerable attention [16,17].

Accordingly, this study aimed to evaluate the effects of chickpea powder (CPP), prepared using oven-drying and freeze-drying methods, at various inclusion levels by (1) analyzing the rheological properties of pork MP after its addition and (2) assessing its impact on the quality of low-fat model sausages (LFMSs) when used as a fat replacer.

## MATERIALS AND METHODS

### Materials

Pork loin and ham were purchased from a pork distribution company in Buk-Gu, Gwangju, South Korea (three-way crossbreed, Landrace×Yorkshire×Duroc, first grade). External fat and connective tissue were removed from both the loin and ham. The loin was cut into 1–2-cm^3^ cubes, vacuum-packed in 200-g portions, and stored at −20°C until further analysis for MP extraction. The ham was minced using a meat grinder (M-12S; Hankook Fuji Industrial), vacuum-packed, and stored at −20°C until use.

Chickpeas were purchased from Hundai Nongsan Agricultural Cooperation and processed into powders via freeze-drying and oven-drying at 60°C. Of the 2 kg of purchased chickpeas, 1 kg was washed under running water, drained as thoroughly as possible, vacuum-packed, and frozen at −70°C for 1 day. The frozen chickpeas were then freeze-dried for approximately 5 days using a freeze-dryer (IlShin Bio Base). For oven-drying, the remaining 1 kg of chickpeas was washed, drained, and dried in an oven dryer set to 60°C. After drying, the chickpeas were ground and sieved through a 500-μm mesh to obtain fine powders. The resulting CPPs were stored at −70°C until use.

### Evaluation of rheological properties of pork myofibrillar protein gel with various levels of chickpea powder processed via different drying methods

#### Preparation of pork myofibrillar protein gel

Frozen pork loin was thawed at 4°C for 12 hours and ground with four volumes of 0.1 M NaCl and 50 mM sodium phosphate buffer for 90 seconds using a mixer. The ground mixture was then centrifuged (Supra 22K; Hanil Science Medical) at 930×g for 15 minutes at 4°C, and the supernatant was discarded to obtain the precipitate (pellet). This process was repeated three times in total. The resulting pellet was mixed with eight volumes of 0.1 M NaCl, filtered through sterile gauze to remove impurities, and centrifuged under the same conditions to extract MP. The pellet was then analyzed for protein concentration using the Biuret method, and the final protein concentration was adjusted to 40 mg/mL. CPPs prepared using different drying methods were incorporated at 0.5%, 1.0%, and 1.5% of the total formulation. The prepared gels were dispensed into vials (Thermo Fisher Scientific) at 5 mL per vial and gradually heated in a water bath (WB-22; Daihan Scientific) from room temperature to 80°C. After heating, the samples were rapidly cooled on ice and stored at 4°C until analysis.

#### Viscosity

The viscosity of the MP gel was measured before heating using a concentric cylinder-type rotational rheometer (RC30; Rheotec Messtechnik). The shear rate was programmed to linearly increase from 0 to 600/s over 360 seconds. Shear rate and shear stress data were plotted using Excel.

#### Cooking yield (%)

Samples stored at 4°C after heating were used for analysis. The inner wall of each vial was scraped and the samples were incubated at room temperature for 1 hour to allow released moisture to accumulate. This process was repeated twice. The total released moisture was measured and applied to the following equation to calculate cooking yield (CY):


(1) CY (%)=(Initial sample weight-Total released moisture weight)/Initial sample weight×100

#### Gel strength (gf)

Samples used for CY measurement were subsequently analyzed for gel strength. Gel strength was determined using a puncture test with the Merlin program on a Universal Testing Machine (3344; Instron Corporation). The crosshead speed was set to 50 mm/min.

#### Protein surface hydrophobicity

Protein surface hydrophobicity was measured according to the method of Chelh et al [18]. A bromophenol blue (BPB) solution containing 1 mg/mL of BPB sodium salt was prepared. A 1-mL MP mixture was reacted with 40 μL of BPB solution (2 mg/mL) for 10 minutes. As a control, 20 mM sodium phosphate buffer (pH 6.0) was used instead of the MP mixture. After centrifugation at 8,000×g for 15 minutes, the absorbance of the supernatant was measured at 595 nm. BPB binding was calculated using the following formula:


(2) $$\text{BPB bound }(\mu \text{g})=40\;\mu \text{g}\times ({\text{A}}_{\text{control}}-{\text{A}}_{\text{sample}})/{\text{A}}_{\text{control}}$$

Where A represents the absorbance result.

#### Sulfhydryl (-SH) and disulfide (S-S) group levels

-SH and S-S group levels (μmol/g protein) were determined using the method described by Cui et al [19] with 5,5’-dithio-bis-(2-nitrobenzoic acid) (DTNB, Sigma-Aldrich). Similar to protein surface hydrophobicity measurement, 0.5 mL and 2.0 mL of the MP mixture (2 mg/mL) were prepared for analysis.

To determine-SH group level, 0.5 mL of each sample was mixed with 2.5 mL of Tris-glycine-8 M urea buffer and 20 μL of DTNB reagent, then incubated for 30 minutes. After centrifugation at 12,000×g for 2 minutes, the absorbance of the supernatant was measured at 412 nm. The -SH group level was calculated using the following equation:


(3) $$-\text{SH group level}=73.53{\text{A}}_{\text{sample}}\times 6.04_{\text{dilution coefficent}}/2_{\text{protein concentration}}$$

Where A represents the absorbance result.

To determine S-S group level, 0.2 mL of the MP mixture was mixed with Tris-glycine-10 M urea buffer and 20 μL of 2-mercaptoethanol, then incubated for 1 hour. Next, 10 mL of 12% trichloroacetic acid were added, and the mixture was incubated at room temperature for 1 hour. After centrifugation at 12,000×g for 2 minutes, the precipitate was washed with cold acetone and reacted with 3 mL of Tris-glycine-8 M urea buffer and 30 μL of DTNB reagent for 30 minutes. After an additional centrifugation step (at 12,000×g for 2 minutes), the absorbance of the supernatant was measured at 412 nm. The S-S group level was calculated using the following equation:


(4) $$\text{S-S group level}=(73.53{\text{A}}_{\text{sample}}\times 15_{\text{dilution coefficient}}/2_{\text{protein concentration}})-\text{SH group level}$$

Where A represents the absorbance result.

#### Sodium dodecyl sulfate–polyacrylamide gel electrophoresis

Sodium dodecyl sulfate–polyacrylamide gel electrophoresis (SDS-PAGE) was performed using a Mini PROTEAN 3 Cell (Bio-Rad Laboratories). The loading sample was prepared by mixing 1% protein with a sample buffer. After loading with a standard protein marker (Model #161-0318; Bio-Rad Laboratories), the proteins were separated at 150 V for approximately 90 minutes. Following separation, the gel was stained with Coomassie Brilliant Blue staining solution for 1 hour and then destained using a destaining solution.

#### Low-vacuum scanning electron microscopy

To investigate three-dimensional structural changes associated with non-meat protein content after heating, scanning electron microscopy (SEM) of the heated MP gel was performed. The samples were trimmed into cubes (approximately 3 mm^3^) and fixed in a 2.5% glutaraldehyde solution at 4°C for 24 hours. After fixation, the samples were washed with 0.1 M sodium phosphate buffer (pH 7.0) for 10 minutes and subsequently immersed in a 1% osmium tetroxide solution for 5 hours. The samples were then washed three times with 0.1 M sodium phosphate buffer (pH 7.0) for 10 minutes per wash. Dehydration was carried out by sequentially increasing the ethanol concentration (50%, 60%, 70%, 80%, 90%, and 100%) at 10-minute intervals, followed by immersion in acetone for final pretreatment. The samples were then dried for approximately 24 hours. The dried samples were gold-coated using an auto sputter coater (Model 108; Cressington Scientific Instruments) and observed under a low-vacuum scanning electron microscope (LV-SEM, JSM-6610LV; JEOL) to examine surface morphology.

### Evaluation of physicochemical and textural properties of low-fat model sausages containing various levels of chickpea powder processed via different drying methods

#### Preparation of low-fat model sausages

LFMSs were prepared according to the formulation in Table 1. Frozen raw meat was thawed at 4°C for approximately 12 hours before use, and soy protein isolate (SPI) used in the reference group (REF) was hydrated in distilled water at a 1:4 ratio (w/w). CPP was prepared using different drying methods and incorporated at varying levels (1.0% and 1.5%). The raw meat and additives were chopped; after chopping, the mixture was packed into 50-mL conical tubes (SPL Life Science) in 40 g portions. Sausages were then heated until the center temperature reached 72°C. When heating was complete, the sausages were rapidly cooled in ice and stored at 4°C until analysis.

#### pH and color values

pH values were measured using a solid-type pH meter (Model 340; Mettler-Toledo). Measurements were taken five times, and the average value was calculated. Color parameters were determined using a Minolta Color Reader (CR-10; Minolta). The cross-sectional surface of the LFMS was measured six times; average values for lightness (CIE L*), redness (CIE a*), and yellowness (CIE b*) were recorded.

#### Proximate analysis (AOAC)

Samples were ground and stored under refrigeration before analysis of proximate composition in accordance with the AOAC International [20] method. Moisture, protein, and fat contents (%) were determined using the dry-oven, Kjeldahl, and Soxhlet extraction methods, respectively.

#### Cooking loss (%)

Cooking loss (CL; %) was determined by measuring sausage weights before and after cooking. The difference was calculated using the following equation, and the average value was reported:


(5) $$\begin{array}{l} \text{CL }(\%)=[\text{Sample weight before cooking }(\text{g})-\text{Sample}\\ \text{weight after cooking }(\text{g})]/\text{Sample weight before cooking}\\ (\text{g})\times 100 \end{array}$$

#### Expressible moisture (%)

Sausage samples were cut into cubic shapes (1.5 g each) for expressible moisture (EM; %) measurement. Prepared samples were wrapped in three layers of filter paper (Whatman #3 filter paper; GE Healthcare), placed in a 50-mL conical tube, and centrifuged at 1,660×g for 15 minutes (VS-5500; Vision Science). The amount of EM absorbed by the filter paper was measured, and EM (%) was calculated using the following equation:


(6) EM (%)=Amount of water absorbed by filter paper (g)/Sample weight (g)×100

#### Texture profile analysis

To evaluate the textural properties of LFMSs, cylindrical samples (diameter: 1.25 cm, height: 1.3 cm) were prepared (10 samples per treatment group). Texture profile analysis (TPA) was conducted using a Universal Testing Machine (3344; Instron Corporation) to measure hardness (gf), springiness (mm), gumminess, chewiness, and cohesiveness. Each sample was analyzed 10 times, and the results were expressed as mean values.

### Statistical analysis

This study was replicated three times (n = 3), and data were analyzed using IBM SPSS software (ver. 29.0; SPSS). One-way analysis of variance was performed for all data except those related to CY (%), gel strength, protein hydrophobicity, and SH and S-S group levels, which were analyzed using two-way analysis of variance, considering two factors: CPP concentration and drying method. Significant differences were determined using Duncan’s multiple range test at p<0.05.

## RESULTS AND DISCUSSION

### Evaluation of rheological properties of pork myofibrillar protein gel containing various levels of chickpea powder processed via different drying methods

#### Viscosity

**Figure 1:**
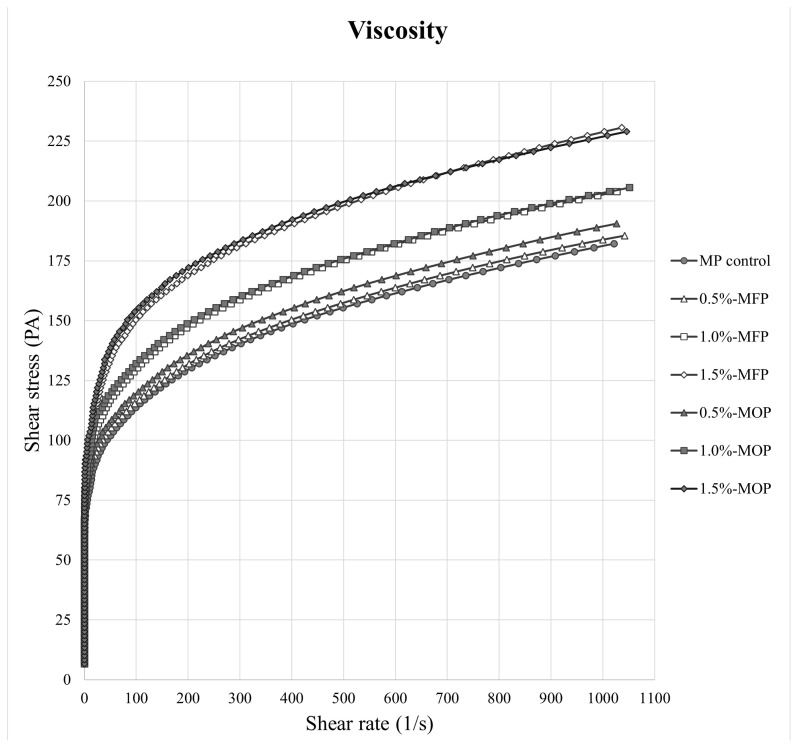
Rheological properties of myofibrillar protein (MP) paste added with different drying methods and various concentrations of chickpea powder (CPP). Treatment: MP, MP control; 0.5%-MFP, MP added with 0.5% freeze-dried CPP; 1.0%-MFP, MP added with 1.0% freeze-dried CPP; 1.5%-MFP, MP added with 1.5% freeze-dried CPP; 0.5%-MOP, MP added with 0.5% oven-dried CPP; 1.0%-MOP, MP added with 1.0% oven-dried CPP; 1.5%-MOP, MP added with 1.5% oven-dried CPP.

The viscosities of MP pastes with varying CPP concentrations processed using different drying methods are presented in Figure 1. In all treatments, shear stress exhibited an increasing trend with rising shear rate. However, the MP control group without CPP showed the lowest shear stress, whereas the addition of CPP generally increased shear stress. Notably, as CPP content increased, viscosity tended to rise. At 0.5% CPP addition, a difference was observed depending on the drying method, such that the oven-dried CPP treatment exhibited higher shear stress than the freeze-dried treatment as shear rate increased. At concentrations other than 0.5%, viscosity remained similar regardless of drying method. Previous studies also revealed an increase in viscosity when MP was supplemented with *Rhynchosia nulubilis* powder and faba bean protein isolate (FBPI) [21,22]. Suchkov et al [23] explained that this phenomenon occurs because globulins in legumes exhibit high viscosity during the crystallization process. Moreover, Zhu et al [24] reported that increasing pea protein isolate (PPI) content in duck myofibrillar protein isolate (DMPI) led to a higher final storage modulus (G′). This phenomenon presumably occurred because PPI facilitated the gelation of DMPI, resulting in a denser gel structure and enhanced gel strength. Therefore, CPP addition may influence protein network formation and alter the physical properties of MP paste.

#### Cooking yield (%) and gel strength (gf)

Table 2 presents the CY (%) and gel strength (gf) of MP gels prepared using CPP processed via different drying methods. As CPP content increased, CY (%) exhibited an increasing trend; a particularly high yield (>90%) was observed after 1.5% CPP addition (p< 0.05). Regarding the effect of drying methods, freeze-dried CPP addition resulted in a higher CY (89.5%) compared with oven-dried treatment (89.0%) (p<0.05), although the numerical difference was minimal. The increase in CY (%) according to CPP content was attributed to the presence of additional chickpea proteins and dietary fibers, which enhance WHC and gel-forming properties. This effect is likely associated with the formation of a more compact gel structure, ultimately contributing to improved water retention. Kim and Chin [21] previously reported that FBPI addition increased the WHC of MP gels. Similarly, the incorporation of non-meat proteins such as chickpea protein isolate (CPI) and red bean protein isolate enhances the WHC of MP gels, emphasizing that plant-based proteins improve water retention in meat products [25,26].

Gel strength (gf) also showed an increasing trend with higher CPP content (p<0.05). However, with respect to drying method, no significant difference in gel strength was observed between the freeze-dried powder (126 gf) and oven-dried powder (128 gf) treatment groups (p>0.05). Li et al [25] reported that the addition of CPI to MP increased protein-protein interactions, resulting in the formation of a denser gel structure upon heating, which significantly enhanced gel strength. Similarly, in the present study, the increase in gel strength according to CPP content was linked to enhanced functionality, consistent with trends observed in previous studies involving legume proteins.

#### Protein surface hydrophobicity

Protein surface hydrophobicity is determined by the amount of hydrophobic amino acids bound to BPB and serves as an indicator for evaluating protein structural changes and denaturation [27]. Table 3 presents the results of protein surface hydrophobicity in MP gels containing CPP processed via different drying methods. As CPP content increased, protein surface hydrophobicity decreased (p<0.05). However, Kim and Chin [21] reported the opposite trend, where FBPI addition increased protein surface hydrophobicity; they explained that the hydrophobic amino acid sequences of 7S and 11S globulins improved surface hydrophobicity by exposing hydrophobic regions and altering protein structure. Conversely, Wu et al [28] observed a decrease in the surface hydrophobicity of duck MP upon addition of kidney bean dietary fiber, attributing this effect to dietary fiber binding to the protein. In the present study, CPP contained both protein and dietary fiber. Thus, when CPP was added to pork MP, dietary fiber likely bound to the MP protein, thereby decreasing surface hydrophobicity and promoting protein aggregation [28,29].

#### -SH and S-S group levels

The levels of -SH and S-S groups exhibited contrasting changes according to CPP content (Table 3). The -SH group level decreased as CPP content increased, whereas the S-S group level began to rise upon addition of 0.5% CPP (p<0.05). Specifically, no significant difference was observed between the MP control and the 0.5% CPP addition, with S-S group levels of 5.14 and 6.36 μmol/g protein, respectively (p>0.05). These results indicate that CPP addition reduced sulfhydryl group content in MP gels and promoted disulfide bond formation. This finding aligns with previous studies that showed similar changes when various non-meat proteins, such as SPI, peanut protein isolate, and FBPI, were incorporated into chicken, red snapper, and pork MPs [21,30,31]. Zhang et al [32] reported that as the -SH groups of actomyosin became more exposed, -S-S group formation activity increased, leading to a reduction in total -SH content. This finding aligns with the results of the present study and suggests that CPP addition positively influences the chemical bonding structure of MP gels. Additionally, Hettiarachchy et al [33] reported that 7S and 11S globulins, the main components of legume proteins, contribute to gel formation within the protein structure and modify intramolecular disulfide bonds.

#### Sodium dodecyl sulfate–polyacrylamide gel electrophoresis

**Figure 2:**
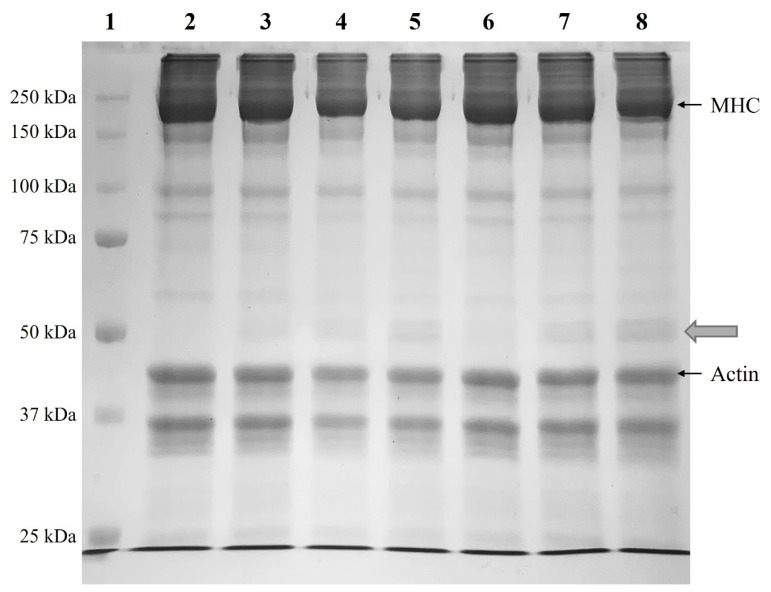
SDS-PAGE patterns of myofibrillar protein (MP) paste added with different drying methods and various concentrations of chickpea powder (CPP). Treatment: 1: STD = standard marker; 2: MP = MP control; 3: 0.5%-MFP = MP added with 0.5% freeze-dried CPP; 4: 1.0%-MFP = MP added with 1.0% freeze-dried CPP; 5: 1.5%-MFP = MP added with 1.5% freeze-dried CPP; 6: 0.5%-MOP = MP added with 0.5% oven-dried CPP; 7: 1.0%-MOP = MP added with 1.0% oven-dried CP; 8: 1.5%-MOP = MP added with 1.5% oven-dried CPP. SDS-PAGE, sodium dodecyl sulfate–polyacrylamide gel electrophoresis.

The SDS-PAGE patterns of MP gels prepared using CPP processed via different drying methods are shown in Figure 2. The main protein bands observed were myosin heavy chain (MHC) and actin, which appeared in the approximate ranges of 250 kDa and 37–50 kDa, respectively. As CPP concentration increased, MHC band intensity decreased, whereas actin band intensity showed minimal change. In addition to the MP control lacking CPP, bands were observed around 50 kDa in the CPP-treated groups, with band intensity increasing according to CPP content. The strongest band intensity was observed in the 1.5% CPP addition group (Lanes 5 and 8), regardless of drying method. Chang et al [34] analyzed the SDS-PAGE patterns of proteins isolated from chickpeas; they identified bands at 15, 34, and 70 kDa as vicilin subunit 7S proteins and bands at 10, 12, and 41 kDa as legumin subunit 11S proteins. Li et al [25] reported that SDS-PAGE results of MP with CPI showed increased band intensity around 50 kDa as CPI content increased, corresponding to vicilin subunit 7S proteins. In the present study, the increase in band intensity around 50 kDa according to CPP content was consistent with these previous findings.

#### Low-vacuum scanning electron microscopy

**Figure 3:**
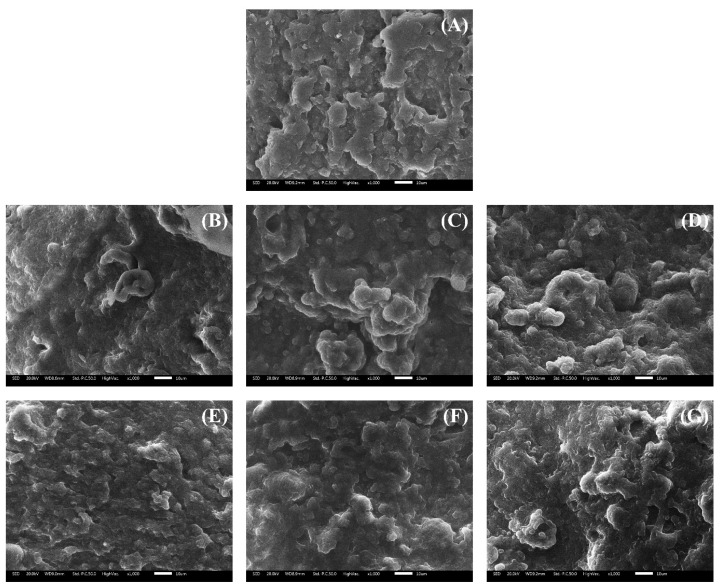
Scanning electron micrographs (×1,000 magnification) of myofibrillar protein (MP) paste added with different drying methods and various concentrations of chickpea powder (CPP). Treatment: (A) MP, MP control; (B) 0.5%-MFP, MP added with 0.5% freeze-dried CPP; (C) 1.0%-MFP, MP added with 1.0% freeze-dried CPP; (D) 1.5%-MFP, MP added with 1.5% freeze-dried CPP; (E) 0.5%-MOP, MP added with 0.5% oven-dried CPP; (F) 1.0%-MOP, MP added with 1.0% oven-dried CPP; (G) 1.5%-MOP, MP added with 1.5% oven-dried CPP.

Figure 3 illustrates the three-dimensional surface microstructure of MP gels prepared using CPP processed via different drying methods. As shown in Figure 3A, the surface of the MP control exhibited relatively irregular and large pores with rough cross-linking. However, as CPP content increased, pore size decreased, the surface became more compact, and particles were more tightly bound. These findings align with previous studies. Li et al [25] reported that increasing CPI content resulted in a denser and more uniform surface while reducing the porosity of the gel structure. The enhanced compactness and refined microstructure observed with increasing CPP content in this study are likely due to cross-linking between protein residues and the reinforcement of covalent bonding.

### Evaluation of physicochemical and textural properties of low-fat model sausages containing various levels of chickpea powder processed via different drying methods

#### pH and color values

pH is a critical factor influencing meat product quality, particularly in relation to color, tenderness, flavor, WHC, and shelf life [35]. As freeze-dried CPP content increased, the pH values of LFMSs also increased from 6.11 to 6.13 (p<0.05). However, in the oven-dried CPP treatment, the pH value remained constant at 6.12, regardless of amount added (p>0.05) (Table 4). Furthermore, regardless of drying method, pH values ranged from 6.11 to 6.13 and did not significantly differ (p>0.05). The pH value of REF was the highest at 6.15, suggesting that addition of SPI and CPP influenced the pH of meat products. Similarly, Kandil et al [36] reported that when CPI was used as a partial fat replacer, the pH of beef sausages increased to a range of 6.6–6.8, higher than the range within the control.

As shown in Table 4, no significant differences in L* values were observed among treatments (p>0.05). However, the addition of non-meat proteins affected the a* and b* values of LFMSs (p<0.05). Regarding a* values, CTL showed the highest value at 10.0; regardless of drying method, the a* value decreased as CPP content increased (p<0.05). The a* value of REF was 9.66, similar to that of LFMS with 1.0% CPP (p>0.05). In contrast, the b* value exhibited an opposite trend to the a* value. The b* value of CTL was the lowest at 6.55 (p<0.05), but no significant differences in b* values were observed among CPP-treated groups (p<0.05). Additionally, no significant differences were found between the b* value of REF and those of all CPP-treated groups (p>0.05). Sanjeewa et al [7] reported that as CPP content increased, the b* value of a low-fat bologna model system also increased. Similarly, Dzudie et al [37] found that when common bean flour was added at levels above 7.5% in sausages, color values differed from those of the control, with a decrease in redness (a*) and an increase in yellowness (b*). Conversely, studies by Choi and Chin [38] and Kim and Chin [21] revealed that the addition of PPI and FBPI did not result in significant changes in sausage color. Therefore, the effects of legume proteins on meat product color may according to type, state, and concentration.

#### Proximate analysis (AOAC)

The proximate composition results of LFMSs containing various CPP concentrations processed via different drying methods are shown in Table 5. The moisture and fat contents of LFMSs ranged from 77.3% to 79.0% and 2.26% to 2.82%, respectively; there were no significant differences among treatments (p>0.05). Conversely, significant differences in protein content were observed among LFMS treatments; REF exhibited the highest protein content at 17.4%, whereas CTL had the lowest at 15.5% (p<0.05). Regardless of drying method, protein content increased with increasing CPP addition (p<0.05). Previous studies have demonstrated that CPI incorporation into meat products reduces moisture content, thereby increasing the relative concentrations of other nutrients (Kandil et al [36]).

#### Cooking loss and expressible moisture (%)

**Figure 4:**
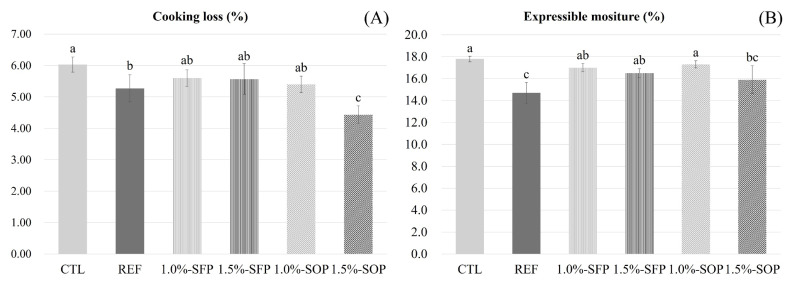
Cooking loss and expressible moisture of low-fat model sausages (LFMSs) added with chickpea powders (CPP) processed by different drying methods and concetrations. (A) Cooking loss (%); (B) Expressible moisture (%). Treatment: CTL, LFMS; REF, LFMS added with 1.5% soy protein isolate; 1.0%-SFP, LFMS added with 1.0% freeze-dried CPP; 1.5%-SFP, LFMS added with 1.5% freeze-dried CPP;1.0%-SOP, LFMS added with 1.0% oven-dried CPP; 1.5%-SOP, LFMS added with 1.5% oven-dried CPP. ^a–c^ Means values with different small letters differ according to the different drying methods and various concentrations of chickpea powders (p<0.05).

Figure 4 shows the CL and EM (%) of LFMSs containing various CPP concentrations processed via different drying methods. As oven-dried CPP concentration increased, CL (%) decreased; 1.5%-SOP exhibited the lowest CL at 4.43%, which was significantly lower than that of CTL (6.03%) and REF (5.27%) (p<0.05). In contrast, for freeze-dried CPP, CL remained similar regardless of concentration (values were 5.60% and 5.57%); no significant differences were detected relative to CTL and REF (p>0.05). Notably, when 1.5% CPP was incorporated, a significant difference in CL was observed between drying methods, indicating that oven-dried CPP was more effective in reducing CL than freeze-dried CPP (p<0.05).

Figure 4B presents the results for EM (%), revealing a trend similar to that of CL (%). As oven-dried CPP concentration increased, EM decreased (17.3% and 15.9%) to values lower than that of CTL (17.8%) (p<0.05). In particular, 1.5%-SOP (15.9%) exhibited EM similar to that of REF (14.7%) (p>0.05). In contrast, for freeze-dried CPP, regardless of concentration (1.0%-SFP: 17.0%, 1.5%-SFP: 16.5%), no significant difference was observed relative to CTL, and no difference between drying methods was detected at the same concentration (p>0.05). Sanjeewa et al [7] reported that CPP supplementation could help to reduce EM in low-fat bologna, although this effect may vary according to the composition of the additive (protein and moisture content). Similarly, the present findings suggest that CPP supplementation influences EM regulation in the product.

#### Texture profile analysis

According to the results presented in Table 5, when CPP was added at concentrations of 1.0% and 1.5%, both oven-dried and freeze-dried, the hardness and chewiness properties of LFMSs increased with higher concentrations (p<0.05). In contrast, no significant changes were observed in springiness, gumminess, or cohesiveness due to differences in concentration or drying method (p>0.05). REF exhibited the highest hardness value at 4005 gf; CTL, which did not contain legume protein, had the lowest value at 2733 gf (p<0.05). Among CPP-supplemented treatments, regardless of drying method, hardness values at 1.5% were 3,738 gf and 3,745 gf, respectively; these were comparable to the value of REF (p>0.05). However, the increase in hardness was less pronounced at 1.0% than at 1.5% (p>0.05). These findings imply that CPP concentration influences the physical properties of the sausages. Yeater et al [39] reported that the inclusion of non-meat proteins in meat products induces changes in textural properties because higher protein content contributes to protein gelation, leading to increased hardness. It has also been suggested that non-meat ingredients promote gel network formation, thereby improving texture, WHC, and molecular forces of meat proteins [40]. Kim and Chin [21] observed that the addition of FBPI increased the hardness of low-fat pork sausages, whereas Sanjeewa et al [7] reported that 5.0% CPP supplementation enhanced sausage hardness, likely because the protein content of the additive contributed to texture improvement.

#### Sodium dodecyl sulfate–polyacrylamide gel electrophoresis

**Figure 5:**
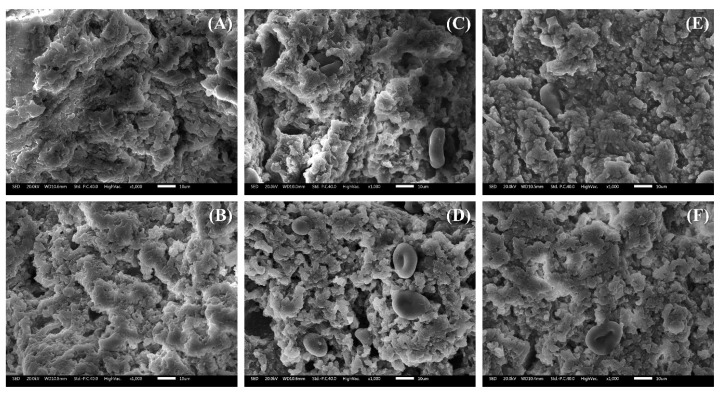
Scanning electron micrographs (×1,000 magnification) of low-fat model sausages (LFMS) added with different drying methods and various concentrations of chickpea powders (CPP). Treatment: (A) CTL, LFMS; (B) REF, LFMS added with 1.5% soy protein isolate; (C) 1.0%-SFP, LFMS added with 1.0% freeze-dried CPP; (D) 1.5%-SFP, LFMS added with 1.5% freeze-dried CPP; (E) 1.0%-SOP, LFMS added with 1.0% oven-dried CPP; (F) 1.5%-SOP, LFMS added with 1.5% oven-dried CPP.

The SDS-PAGE patterns of LFMSs formulated with different concentrations of oven-dried and freeze-dried CPP before and after cooking are presented in Figure 5. In all treatments, both before and after cooking, bands corresponding to MHC (~250 kDa) and actin (~50 kDa) were observed; there were no noticeable differences among treatments. After cooking, all samples exhibited high-molecular-weight biopolymer bands, which were particularly intensified in treatments containing CPP. The intensity of these high-molecular-weight bands increased with CPP concentration and was comparable to that of REF supplemented with SPI. These results suggest that CPP addition promotes protein polymer formation during the thermal processing of LFMSs, contributing to improved functional and structural properties.

#### Low-vacuum scanning electron microscopy

**Figure 6:**
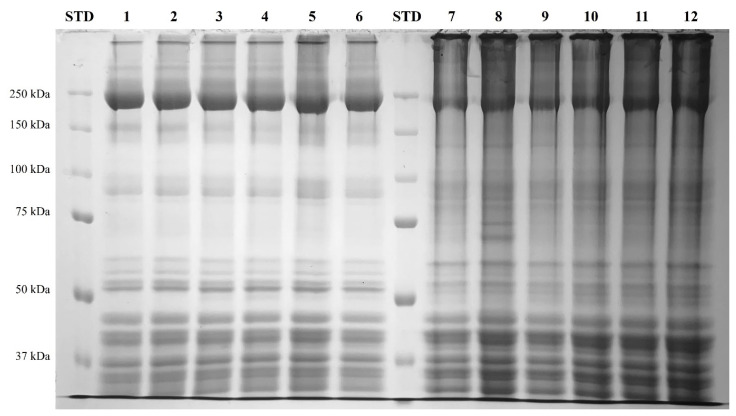
SDS-PAGE patterns of low-fat model sausages (LFMS) added with different drying methods and various concentrations of chickpea powders (CPP). STD: standard marker. Lane 1–6: before cooking (batter), Lane 7–12: after cooking (sausage) (Lane 1 and 7: CTL, LFMS; Lane 2 and 8: REF, LFMS added with 1.5% soy protein isolate; Lane 3 and 9: 1.0%-SFP, LFMS added with 1.0% freeze-dried CPP; Lane 4 and 10: 1.5%-SFP, LFMS added with 1.5% freeze-dried CPP; Lane 5 and 11 1.0%-SOP, LFMS added with 1.0% oven-dried CPP; Lane 6 and 12: 1.5%-SOP, LFMS added with 1.5% oven-dried CPP).

Figure 6 presents the microstructure of LFMSs containing various CPP concentrations processed via different drying methods. CTL (A) exhibited multiple pores on the surface, likely due to the expansion or movement of water and air trapped within the gel matrix during sausage processing [41,42]. Lu et al [43] reported that such internal pores serve as pathways for water loss, leading to reduced CY and WHC, ultimately limiting the ability of sausage to retain water. REF (B) displayed a denser and more compact network with fewer pores relative to CTL. In treatments with CPP (C–F), an increasing concentration resulted in a more uniform and compact surface with smaller pore sizes. According to Zhao et al [35], a dense gel network structure with numerous small pores can help limit water loss and improve gel characteristics. Accordingly, treatment with 1.5% CPP exhibited reduced CL and EM content while increasing yield and positively influencing texture. These results are consistent with the findings presented in Figure 4 and Table 4.

## CONCLUSION

As CPP concentration increased, the viscosity, CY, and gel strength of MP gels significantly improved; protein surface hydrophobicity and -SH group levels decreased, whereas S-S group levels increased, indicating enhanced protein-protein interactions and network structure formation. Additionally, SDS-PAGE and SEM analyses demonstrated that CPP addition improved the structural and functional properties of MP and LFMS. In LFMSs, higher CPP concentrations had beneficial effects, including reductions in CL and EM content and an improvement in texture; the best quality characteristics were observed in the 1.5% oven-dried CPP treatment. These findings suggest that CPP can help to improve meat product quality as a fat replacer and functional protein ingredient. Future studies should further evaluate the sensory properties and consumer preferences of meat products containing CPP to assess their potential in commercial applications.

## Figures and Tables

**Table 1:** Formulation of low-fat model sausages (LFMSs) with different drying methods and various levels of chickpea powder (CPP)

Ingredients (%)	Treatment^1)^					

	CTL	REF	1.0%-SFP	1.5%-SFP	1.0%-SOP	1.5%-SOP
Meat	70.00	70.00	70.00	70.00	70.00	70.00
Water	28.13	28.13	28.13	28.13	28.13	28.13
Ice water	28.13	12.13	28.13	28.13	28.13	28.13
Hydrate water	0.00	16.00	0.00	0.00	0.00	0.00
NMI	1.87	3.37	2.87	3.37	2.87	3.37
Salt	1.50	1.50	1.50	1.50	1.50	1.50
STPP	0.30	0.30	0.30	0.30	0.30	0.30
Sodium erythorbate	0.05	0.05	0.05	0.05	0.05	0.05
Sodium nitrite	0.015	0.015	0.015	0.015	0.015	0.015
Soy protein isolate	0.00	1.50	0.00	0.00	0.00	0.00
Chickpea powder	0.00	0.00	1.00	1.50	1.00	1.50
Freeze-drying	0.00	0.00	1.00	1.50	0.00	0.00
Oven drying	0.00	0.00	0.00	0.00	1.00	1.50
Total	100.00	101.50	101.00	101.50	101.00	101.50

1) Treatment: CTL, LFMS; REF, LFMS with 1.5% soy protein isolate; 1.0%-SFP, LFMS with 1.0% freeze-dried CPP; 1.5%-SFP, LFMS with 1.5% freeze-dried CPP; 1.0%-SOP, LFMS with 1.0% oven-dried CPP; 1.5%-SOP, LFMS with 1.5% oven-dried CPP.

NMI, non-meat ingredients; STPP, sodium tripolyphosphate.

**Table 2:** Cooking yield (%) and gel strength (gf) of myofibrillar protein (MP) paste with different drying methods and various levels of chickpea powder (CPP)

		Cooking yield (%)	Gel strength (gf)
Concentration treatment^1)^	0%	86.4±0.30^d^	108±5.71^d^
	0.5%	89.0±0.26^c^	122±5.50^c^
	1.0%	90.4±0.43^b^	133±6.82^b^
	1.5%	91.3±0.45^a^	144±9.44^a^
Drying treatment^2)^	Freeze	89.5±2.10^A^	126±16.0^A^
	Oven	89.0±1.79^B^	128±15.2^A^

1) Concentration treatment: 0%, MP control; 0.5%, MP with 0.5% CPP; 1.0%, MP with 1.0% CPP; 1.5%, MP with 1.5% CPP.

2) Drying treatment: Freeze, MP with freeze-dried CPP; Oven, MP with oven-dried CPP.

A,B Mean values with different superscripts differ according to CPP drying method (p<0.05).

a–d Mean values with different superscripts differ according to CPP concentration (p<0.05).

**Table 3:** Protein surface hydrophobicity and levels of -SH and -S-S groups in myofibrillar protein (MP) paste with different drying methods and various levels of chickpea powder (CPP)

		Hydrophobicity (Bound BPB, μg)	-SH group level (μmol/g protein)	S-S group level (μmol/g protein)
Concentration treatment^1)^	0%	6.06±0.31^a^	95.2±0.10^a^	5.14±0.46^c^
	0.5%	4.72±0.63^b^	85.6±3.33^b^	6.36±0.76^c^
	1.0%	3.89±0.25^c^	78.8±1.66^c^	21.3±2.14^b^
	1.5%	2.71±0.30^d^	70.7±3.68^d^	35.2±1.98^a^
Drying treatment^2)^	Freeze	4.28±1.37^A^	81.7±10.1^A^	17.4±13.3^A^
	Oven	4.40±1.29^A^	83.5±9.19^A^	16.6±12.5^A^

1) Concentration treatment: 0%, MP control; 0.5%, MP with 0.5% CPP; 1.0%, MP with 1.0% CPP; 1.5%, MP with 1.5% CPP.

2) Drying treatment: Freeze, MP with freeze-dried CPP; Oven, MP with oven-dried CPP.

A Mean values with the same superscript in the same parameter do not differ (p>0.05).

a–d Mean values with different superscripts differ according to CPP concentration (p<0.05).

BPB, bromophenol blue.

**Table 4:** pH and color values of low-fat model sausages (LFMSs) with different drying methods and various concentrations of chickpea powder (CPP)

	Treatment^1)^					

	CTL	REF	1.0%-SFP	1.5%-SFP	1.0%-SOP	1.5%-SOP
pH	6.08±0.01^d^	6.15±0.01^a^	6.11±0.01^c^	6.13±0.02^b^	6.12±0.01^bc^	6.12±0.00^bc^
CIE L* (lightness)	69.7±0.34^a^	69.3±0.23^a^	69.5±0.24^a^	69.8±0.23^a^	69.5±0.27^a^	69.8±0.06^a^
CIE a* (redness)	10.0±0.26^a^	9.66±0.08^b^	9.47±0.02^b^	8.86±0.26^c^	9.32±0.16^b^	8.87±0.23^c^
CIE b* (yellowness)	6.55±0.42^c^	7.31±0.31^ab^	7.39±0.23^ab^	7.64±0.19^a^	7.12±0.07^b^	7.39±0.21^ab^

1) Treatment: CTL, LFMS; REF, LFMS with 1.5% soy protein isolate; 1.0%-SFP, LFMS with 1.0% freeze-dried CPP; 1.5%-SFP, LFMS with 1.5% freeze-dried CPP; 1.0%-SOP, LFMS with 1.0% oven-dried CPP; 1.5%-SOP, LFMS with 1.5% oven-dried CPP.

a–d Mean values with different superscripts differ according to drying method and CPP concentration (p<0.05).

**Table 5:** Proximate analysis and texture profile analysis of low-fat model sausages (LFMSs) with different drying methods and various concentrations of chickpea powder (CPP)

	Treatment^1)^					

	CTL	REF	1.0%-SFP	1.5%-SFP	1.0%-SOP	1.5%-SOP
Moisture (%)	78.2±2.23^a^	79.0±1.38^a^	77.5±1.58^a^	76.9±2.20^a^	77.5±1.74^a^	77.3±2.32^a^
Fat (%)	2.49±0.12^a^	2.26±0.33^a^	2.48±0.34^a^	2.82±0.51^a^	2.42±0.27^a^	2.42±0.24^a^
Protein (%)	15.6±0.21^d^	17.4±.15^a^	16.4±0.25^c^	16.8±0.15^b^	16.4±0.15^c^	16.8±0.10^bc^
Hardness (gf)	2,733±112^c^	4,005±69^a^	3,339±172^b^	3,738±214^a^	3,349±175^b^	3,745±280^a^
Springiness (mm)	4.94±0.20^a^	4.92±0.27^a^	4.59±0.18^a^	4.47±0.37^a^	4.69±0.47^a^	4.73±0.06^a^
Gumminess	23.6±1.09^b^	34.7±0.44^a^	30.8±2.21^a^	33.8±5.78^a^	29.9±4.89^a^	34.1±2.49^a^
Chewiness	117±7.47^d^	170±8.70^a^	141±5.10^bc^	150±15.6^abc^	138±9.06^c^	159±15.5^ab^
Cohesiveness	0.87±0.02^a^	0.87±0.03^a^	0.93±0.07^a^	0.90±0.11^a^	0.89±0.12^a^	0.92±0.04^a^

1) Treatment: CTL, LFMS; REF, LFMS with 1.5% soy protein isolate; 1.0%-SFP, LFMS with 1.0% freeze-dried CPP; 1.5%-SFP, LFMS with 1.5% freeze-dried CPP; 1.0%-SOP, LFMS with 1.0% oven-dried CPP; 1.5%-SOP, LFMS with 1.5% oven-dried CPP.

a–d Mean values with different superscripts differ according to drying method and CPP concentration (p<0.05).
